# An emerging interface between life science and nanotechnology: present status and prospects of reproductive healthcare aided by nano-biotechnology

**DOI:** 10.3402/nano.v5.22762

**Published:** 2014-02-26

**Authors:** Rakhi K. Jha, Pradeep K. Jha, Koel Chaudhury, Suresh V.S. Rana, Sujoy K. Guha

**Affiliations:** 1 School of Medical Science and Technology, Indian Institute of Technology Kharagpur, India; 2 Toxicology Laboratory, CCS University, Meerut, India

**Keywords:** nanotechnology, nanomaterials, reproductive healthcare, reproductive organ cancer, fertility control, infertility

## Abstract

Among the various applications of nano-biotechnology, healthcare is considered one of the most significant domains. For that possibility to synthesize various kind of nanoparticles (NPs) and the ever-increasing ability to control their size as well as structure, to improve surface characteristics and binding NPs with other desired curing agents has played an important role. In this paper, a brief sketch of various kinds of nanomaterials and their biomedical applications is given. Despite claims of bio-nanotechnology about to touch all areas of medical science, information pertaining to the role of nanotechnology for the betterment of reproductive healthcare is indeed limited. Therefore, the various achievements of nano-biotechnology for healthcare in general have been illustrated while giving special insight into the role of nano-biotechnology for the future of reproductive healthcare betterment as well as current achievements of nanoscience and nanotechnology in this arena.

It has been witnessed in recent years that nanotechnology has immense potential impact on healthcare and pervades many aspects of a new era aptly labeled ‘nanomedicine’ (1). Equally, it has generated safety concerns both among the scientific community and the public at large. The EU Technology defines nanomedicine as ‘the application of nanotechnology to achieve breakthroughs in healthcare’ (2). It exploits the improved and often physical, chemical, and biological properties of materials at the nanometer scale (3).

Novel properties that differentiate nanomaterials from bulk materials generally develop at a length scale of <100 nm. However, the size at which materials display different properties to the bulk material is material dependent (4) and can certainly be claimed for many materials at size >100 nm as per Malvern guidelines of 2010. From the biological point of view, nanomaterials match the typical size of naturally occurring functional units or components of living organisms and, for this reason, enable more effective interaction with biological systems. The application of nanomaterials in medicine and enhancing quality of life can be understood from state of the art knowledge on nanoscale features of biological systems in order to learn how to design nanodevices for biomedical uses (5). While trying to create something in a nanoscale range, one must notice the well-known biological things in various nano-ranges or micro-ranges (see Table 1). Nanomaterials have a relatively larger surface area and, therefore, are more chemically reactive. In addition, the nano-scale has a marked effect on the strength and electrical properties as the quantum effects dominate the behavior of materials with respect to their optical, electrical, and magnetic properties (6).

**Table 1:** Biological nanoscales with respective natural as well as manmade things in that range

Size range	Natural things	Synthetic things in that range
5,000,000 nm	Ants	Head of a pin (2 mm), grain of salt
2,00,000 nm	Duet mite	Sand grains
10,000–1,00,000 nm	Human hair, pollen, cancer cell	Sheet of paper
10,000–20,000 nm	Fly ash, kidney excretions	Polymeric nanoparticles
1,000–10,000 nm	Cell	MEMS devices
2,000–2,500 nm	*E. coli*, red blood cells	Nanomedicine
100–200 nm	Virus (T4 bacteriophage)	X-ray lens, STM tip
2–20 nm	Ribosome	QDs, nanopores, nanoshells
10 nm	ATP synthatase, nucleic acids (tRNA)	Computer chip, single transistor
4–10 nm	Proteins (chymotrypsin), antibody, large molecules	Dendrimers, plastics
1–2 nm	DNA, glucose, small molecules	Nanotubes, QDs
0.1 nm	Atom, water	–

nm, nanometer; mm, millimeter; QDs: quantum dot.

Basically, nanomaterials fall into three categories: one-, two-, and three-dimensional. Three-dimensional nanomaterials like carbon nanotubes (CNTs) have generated considerable interest, and a significant amount of research was done during the past decade on their potential biomedical applications (7, 8). Boron nitride nanotubes (BNNT) also generated immense curiosity in view of their piezo-electric properties through which they are able to acquire an electric charge on exposure to ultrasound and polarized light (9). Superparamagnetic iron oxide particles (SPIONs) have been the standard contrast agent for magnetic resonance imaging of tumors since the early 1990s. SPIONs coated with dextran are already in established clinical use (10).

Sexual and reproductive health has been defined by the international community as a state of complete physical, mental, and social wellbeing, and not just merely the absence of disease or infirmity, in all matters relating to the reproductive system and to its functions and processes (11). It is an essential component of young people’s ability to become well-adjusted, responsible, and productive members of society as well as quality of life of elders (12).

Our group at IIT Kharagpur has been working on several reproductive healthcare applications of nanotechnology. For example, a novel fertility control polymeric nanocomposite iron oxide–copper–styrene maleic anhydride–dimethyl sulfoxide (Fe_3_O_4_–Cu–SMA–DMSO) tentatively named ‘Smart RISUG’ (Reversible Inhibition of Sperm Under Guidance), in the presence of external pulsed electromagnetic field, can be transported into reproductive tube, monitored externally, and its biodistribution can be controlled and, finally it can be reversed non-invasively for restoration of fertility (13–15) when desired. None of these require surgical intervention due to the presence of magnetic and electric nanoparticles (NPs), and the contraceptive property is imparted due to antimicrobial, one-time injectable, long-term effective molecule SMA (16, 17).

Although current literature claims that nanotechnology is going to play a big role in various arenas of healthcare, information especially pertaining to reproductive healthcare is lacking. Therefore, in this paper a review status of achievements of nanoscience and nano-biotechnology in the area of healthcare (Fig. 1) practically available today is presented with special emphasis placed on the potential role of nanotechnology for various aspects of reproductive healthcare, and additional future possibilities are put forth.

**Fig. 1:**
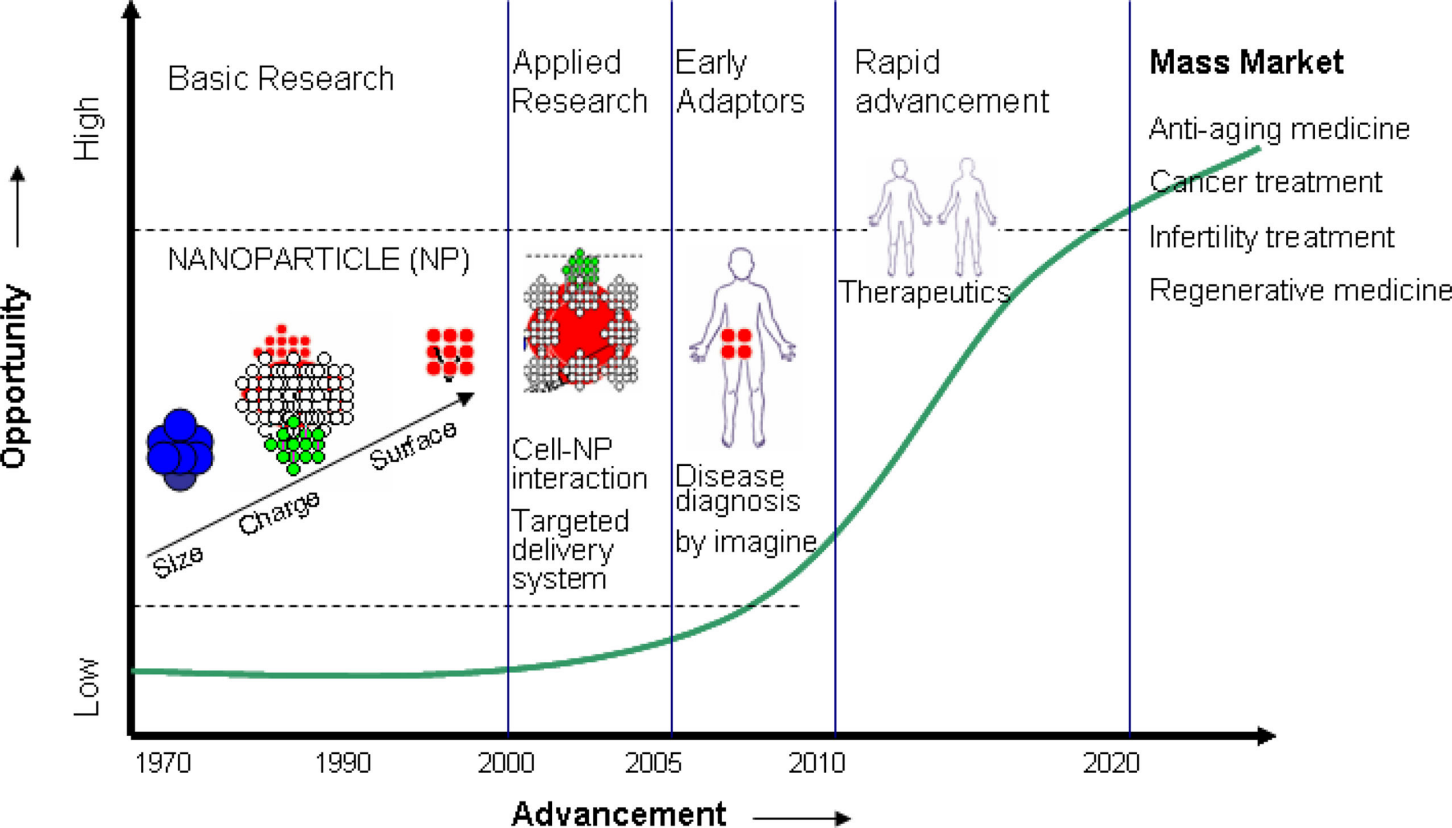
Trajectory of nanotechnology advancement over the years.

## 1. Biocompatible nanomaterials promising for healthcare applications

In recent times, the focus of nanoscience and nanotechnology research has gradually shifted from the development of high-quality nanomaterials and investigation of their properties to application side. Although biomedical science has been recognized as a field that can greatly benefit from nanotechnology, not all of the nanomaterials are suitable for all healthcare applications. Some of the nanomaterial-based drugs, devices have already entered the market and others are on the verge of doing so. A description of nanomaterials found biocompatible for biomedical application is given in Table 2 (6, 13–15, 18–35). Furthermore, a general classification of tools and technologies of nano-biotechnology in healthcare is discussed in the following sections.

**Table 2:** Examples of biocompatible nanomaterials promising for healthcare application

S. No.	Biocompatible nanoparticles (NPs)	Respective nanomedicine	Biomedical applications	Key properties	References
Metallic					
1.	Iron oxide	Polymer nanocomposite Fe_3_O_4_–Cu–SMA–DMSO	Male and female long-term contraception	Magnetic field mediated targeted drug delivery, control of biodistribution, non-invasive imaging and reversibility	(15–18)
		Feridex	MRI contrast	Targets liver	(18)
		NanoTherm	Cancer therapy	Acts against cancerous cells	(19)
		Iron-Platinum alloy nanoparticles	MRI interventional catheter and guidewire	Diagnostic and therapeutic contrast agent, Semi active resonant markers for catheter and passive markers for guidewires	(20)
2.	Gold	Verigene	*In vitro* diagnostics	Genetic	(21)
		Nanogold or colloidal gold	Drug delivery, Biomedical imaging and diagnostics tests	Tunable optical and electronic properties	(22)
		Aurimmune	Cancer therapy	Acts against cancerous cells	(23)
3.	AIE-active fluorogen-loaded BSA NPs	Fluorogen, 2-(2,6-bis((*E*)-4-(phenyl(4′-(1,2,2-triphenylvinyl)-[1,1′-biphenyl]-4-yl)amino)styryl)-4*H*-pyran-4-ylidene)malononitrile (TPE-TPA-DCM)	*In vivo* and *in vitro* imaging, excellent cancer cell uptake	Enhanced permeability and retention effect	(24)
4.	Nanoshell	Auroshell	Auroshell	Targets head, neck	(25)
Semiconductor					
5.	Quantum dots	Qdots, EviTags	*In vitro* diagnostics	Targets tumor cells	(26)
6.	Semiconductor	Nanoco, CrystalPlex, cytodiagnostics	Fluorescence contrast	Acts at molecular level on tissues	(27)
7.	Nanocrystals	Sensors	Contrast agent	Sensing structures	(6)
Organic					
8.	Cyanine dyes	Quantum dots-protein-dye conjugates	*In vivo* NIR fluorophores and FRET imaging could have far-reaching application in optical imaging	Tuning the degree of spectral overlap between donor and acceptor provides unique configuration	(28)
9.	Self-assembled chitosan (CHI) and modified lecithin (ML)	Biocompatible stable nanoparticles	Numerous application like reversible hemostatic action in wounds, drug delivery carriers	Stable over an extended pH and ionic strength range 8.7–67.2% encapsulation efficiency, ability to be converted to lyophilized powder or concentrated suspension.	(29)
10.	Targeted polymer nanoparticles loaded with (−)-epigallocatechin 3-gallate (EGCG)	Chemotherapeutic agent	Powerful potential to prevent prostate cancer (PCa)	Target prostate-specific membrane antigen (PSMA)	(30)
11.	Organically modified silica nanoparticles	Biocompatible nanoparticles	*In vivo* neuron targeting without harming whole organism or causing neuronal death	Penetrate into living brains, neuronal cell bodies and axonal projections without affecting viability	(31)
12.	Polydopamine fluorescent organic nanoparticles	Biocompatible nanoparticles	Cell imaging	Tunable photoluminescence	(25)
13.	5-Fluorouracil (5-FU) loaded biocompatible fluorescent zein nanoparticles	Solid lipid nanoparticles	For simultaneous bioimaging and drug delivery application	Better controlled release kinetics, improved stability, enhanced drug entrapment	(32)
14.	Non-steroidal anti-inflammatory (NSAIDs)-loaded nanoparticles	Biocompatible drug loaded nanoparticles	Models to be further integrated in a prosthesis surface functionalization	Controlled drug release	(33)
15.	Polymeric nanoparticles (NPs)	Biocompatible NPs with therapeutic effect	Potential co-delivery of therapeutic agents	Controlled drug delivery, acid degradable	(34)
16.	Polymeric NPs releasing cargo	Therapeutic multifunctional nanoparticles	Drug targeting, controlled release of therapeutic and diagnostic agents	Degrade and release cargo in response to biologically relevant levels of hydrogen peroxide	(35)

### 1.1. Liposomes

**Fig. 2:**
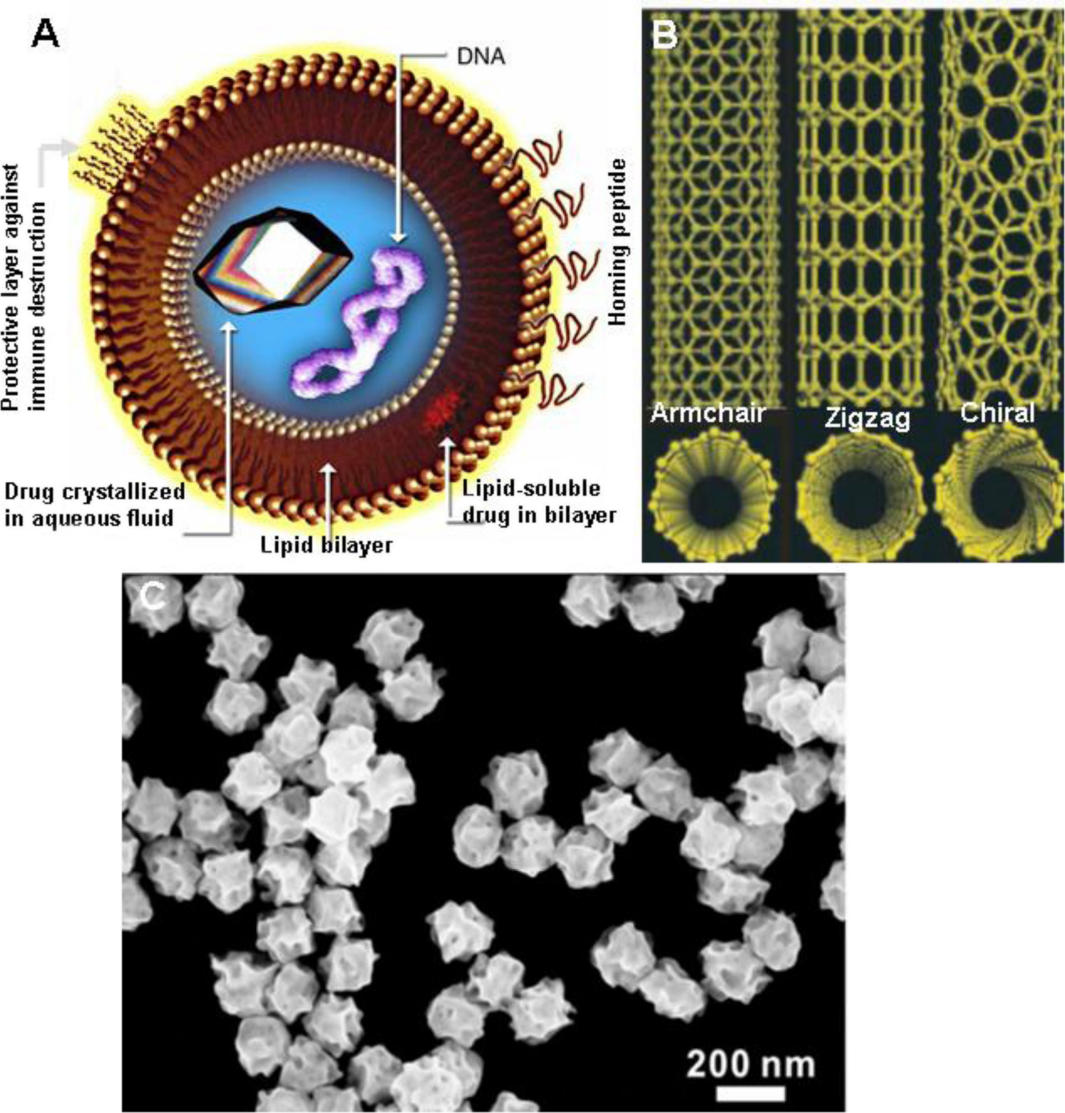
(A) Unilamellar liposomes (Courtesy en.wikipedia.org) (37). (B) Schematic illustrations of carbon nanotube structures of various kind: i. armchair, ii. zigzag, and iii. chiral SWNTs (8). (C) SEM image of gold nanoparticles (AuNPs) with an average size of 189 nm. Reproduced with permission from Zhang et al. 2014 (51).

Liposomes are the hollow balls of lipids – the molecules that form the cell walls of almost every living organism (Fig. 2A) – and were discovered in 1961 by Alec D. Bangham who was studying phospholipids and blood clotting (36, 37). The main component of liposome membranes is dipalmitoyl phosphatidyl choline (DPPC). In principle, liposomes can be prepared using PC only (38). However, some other compounds are added in order to improve stability or other structural properties. Two compounds are generally added: dipalmitoyl phosphatidyl glycerol (DPPG) and cholesterol. Apparently, cholesterol has the effect of making the membrane less permeable by filling up holes or disruptions.

Liposomal doxorubicin (DaunoXome) was first used as a treatment for Kaposi’s sarcoma, a cancer often associated with AIDS (39). Doxorubicin had been around as a cancer drug since the 1960s but its encapsulation in a liposome carrier was new. PEGylated liposomal doxorubicin (Doxil) has shown substantial efficacy in breast cancer treatment both as monotherapy and in combination with other chemotherapeutics. The liposome ball protects the doxorubicin from attack by the body’s immune system ensuring targeted release and prolonged action of the compound at the tumor site. Major challenge with liposome-based drug delivery is the complex biological environment because it involves the interaction of plasma proteins (for example, opsonins) and cells with vesicle surfaces; vesicle size and surface-dependent interception of liposomes by the fixed macrophages of the reticuloendothelial system (RES); penetration of small vesicles through the fenestration of the organ to reach the parenchymal cells; the distribution of these small vesicles into the bone marrow; and to a limited extent extravasation (40).

Liposomal encapsulation technologies (LET) is a particular method for sustained release of additional health supplements that can also solve the problem of bioavailability. Because the system is microscopic and efficient, considerably smaller dosages are needed, thereby conserving medical resources. This way, LET has the real potential to improve healthcare status in a developing country like India because it is efficient, effective, and economical for both the consumer and the producer (41). Magnetic liposomal nanoplatforms for theranostics combine multiple functionalities, including imaging magnetic guidance to the disease site and delivery of drug payload through sustained as well as triggered drug release. *In*-*vivo* multimodal imagings using MRI, SPECT, and FMT using these nanoplatforms have already been demonstrated (42).

### 1.2. Carbon nanotubes

CNTs are usually produced by catalytic chemical vapor deposition and contain metals, chiefly Fe at their closed ends. Therefore, CNTs are paramagnetic, which is a useful property for certain biomedical applications. They have variable diameters (a few nm to 100 nm) and length (up to several mm). Their molecular structure (8) accounts for their unique properties like high tensile strength, high electrical conductivity, heat resistance, efficient thermal conduction, and relative chemical inactivity (Fig. 2B). The exact structure of CNT, especially their n–m, chirality determines their electric properties (43). By virtue of their nano-scale, electron transport in CNTs occurs through quantum effects and thus only propagates uni-dimensionally along the axis of the tube.

CNTs are very prevalent in today’s world of medical research and are being highly researched in the fields of efficient drug delivery and biosensing methods for disease treatment and health monitoring (44). One significant problem that impeded the use of CNTs for biomedical applications, which has since been resolved, is their insolubility in aqueous solution, essential for biological interactions, and biocompatibility. The problem has been resolved by studies on protocols for non-covalent polymer coating, which has enabled *in*-*vitro* cell viability assays and *in vivo* studies on biocompatibility (45, 46).

The other development necessary for biomedical use has been the functionalization of CNTs for carrying drugs, genes, and other biomolecules to target cells and tissues. In Europe, a CNT vector has been developed for gene therapy of certain disorders of the CNS, including stroke. The NINIVE (Non-Invasive Nanotransducer for *In Vivo* gene thErapy) vector offloads its pay load of genes at the disease site on exposure to static electric fields and simultaneously enhances cell permeabilization by a process of CNT-mediated electroporation (47). Resolution of CNT-mediated complement activation that may be related to pro-inflammatory reactions following environmental exposure is largely hindered by the poorly defined surfaces of nanotubes and lack of their reproducible production (48). However, a clear understanding of molecular mechanisms that orchestrate complement activation by both native and surface-modified CNTs will have an impact in the nanotoxicology field.

### 1.3. Metal NPs

NPs can be synthesized through a variety of chemical and physical methods. The choice of preparation procedure depends on the chemical and physical characteristics required in the final product such as size, dispersion, chemical miscibility, optical properties, and so on (49). The range of procedures to prepare metal NPs and films include chemical reduction method, electrochemical, hydrothermal, photochemical, sonochemical, chemical vapor deposition, physical vapor deposition, and so on.

The strong optical absorption and scattering of noble metal NPs is due to an effect called localized surface plasmon resonance (50), which enables the development of novel biomedical applications. Noble metal NPs such as gold, silver, and platinum are particularly of interest due to their size- and shape-dependent unique optoelectronic properties. These noble metal NPs, particularly of gold, have elicited lots of interest for important biomedical applications because of their ease of synthesis, characterization, and surface functionalization (51). Since the manufacture and use of NPs are increasing, humans are more likely to be exposed occupationally or via consumer products and the environment. However, so far toxicity data for most manufactured NPs are limited.

However, the unusual toxicities associated with conventional anti-angiogenic agents (as mentioned previously) may be overcome if these NPs alone can be efficacious as an anti-angiogenic agent. In a landmark study, it was shown that ‘naked’ gold nanoparticles (AuNP) inhibited the activity of heparin-binding proteins, such as VEGF and bFGF *in vitro* and VEGF-induced angiogenesis *in vivo* (52). B-chronic lymphocytic leukemia (B-CLL) is the most widespread form of leukemia. Indeed, B-CLL cells exposed to AuNP exhibited an increase in apoptosis in a dose-dependent manner (53). Historically, gold salts have been used to treat a multitude of inflammatory diseases (Fig. 2C) (54). In a related study, gold beads were implanted near the hip joints of dogs with hip dysplasia in a double-blind clinical trial. Recent innovations in nanotechnology have demonstrated that metallic NPs hold great promise as photodynamic therapy (PDT) and hyperthermic agents. For example, upon X-ray irradiation, AuNP can induce cellular apoptosis through the generation of radicals. This treatment strategy has increased the killing of cancer cells without harming the surrounding healthy tissue (55–57).

### 1.4. Oxide NPs

Preparation methods for metal oxide NPs may be grouped into two main streams based on liquid–solid and gas–solid nature of transformations. Most broadly used methods are liquid–solid transformations that include the co-precipitation method, sol-gel processing, microemulsion technique, solvo-thermal methods, and template/surface derivatized methods. While gas–solid transformation methods are restricted to chemical vapor deposition (CVD) and pulsed laser deposition only (58).

**Fig. 3:**
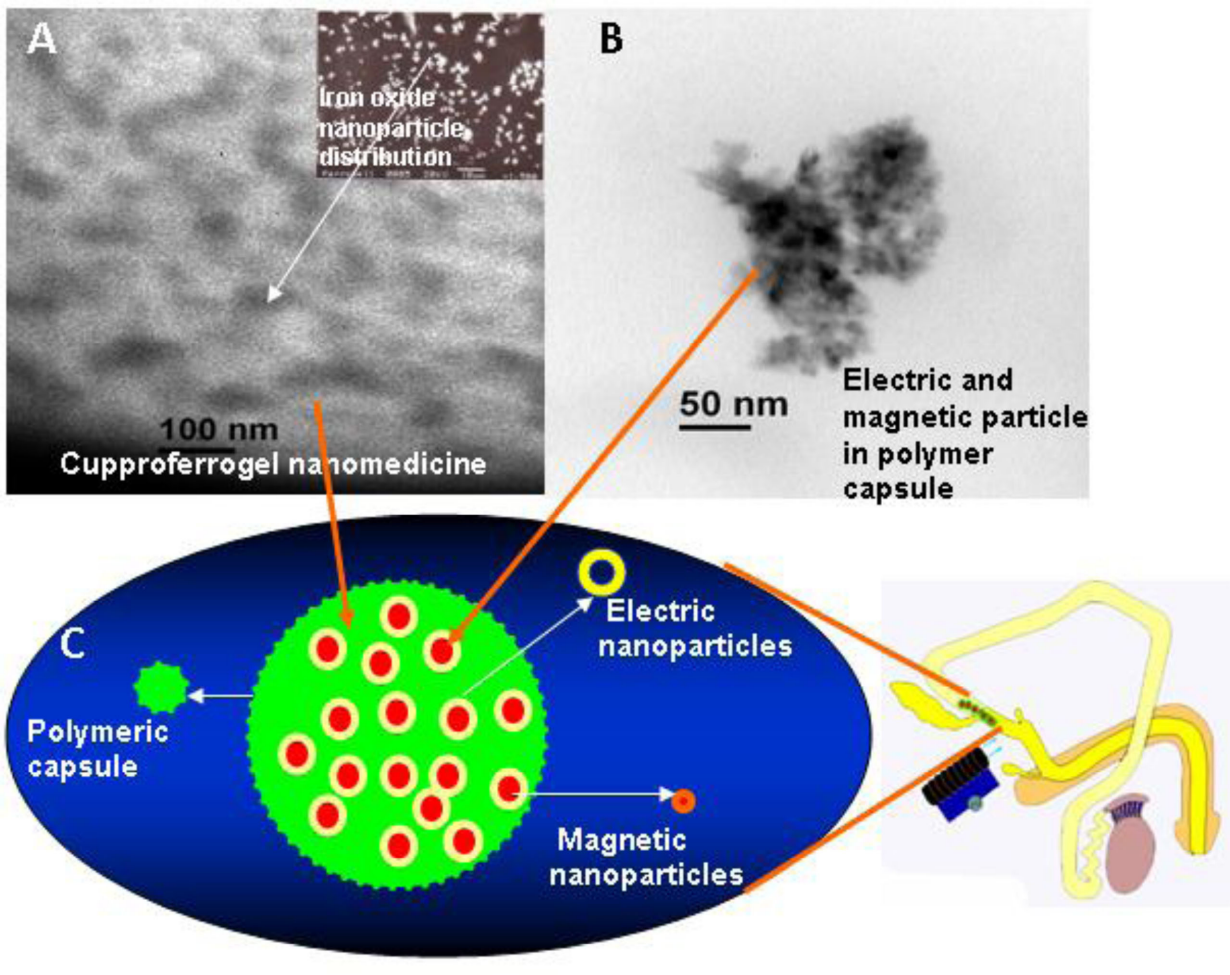
(A) EDS-X-ray microanalysis of the Fe_3_O_4_–Cu–SMA–DMSO (Smart RISUG) indicating arrangement of drug particles in the Cuproferrogel compound. (B) HRTEM of 50–150×10^−9^ m sized Smart RISUG nanoparticles. (C) Schematic representation of Fe_3_O_4_–Cu nanoparticles surrounded by SMA polymer, and its use as a contraceptive.

A bunch of novel applications within these fields rely on the size—dependence of the optical, (electronic and/or ionic) transport, mechanical, and, obviously, surface/chemical (redox, acid/base) properties of oxide nanomaterials. Engineered metal oxide NPs have immense scope for targeted drug delivery, therapeutics, and imaging. For example, iron oxide magnetic NPs in combination with electric particles and a polymer known as ‘Smart RISUG’ developed at IIT Kharagpur lab has shown magnetic field-mediated sperm/ovum interaction (Fig. 3), controlled biodistribution and hence proved as a potential contraceptive as per laboratory studies (13–15). Toxicity studies on the same are in progress in Indian laboratories. Zinc oxide NPs have potential drug delivery applications and are found suitable for the selective destruction of tumor cells (59). Before administering any kind of metal oxide NPs toxicity studies are highly recommended; however, in most studies no measurable effect on cells was detected until concentration reached 200 µg/ml (60).

### 1.5. Carbon nanoparticles

Common routes in making fluorescent carbon nanoparticle (CNP) include the high energy ion beam radiation-based creation of point defect in diamond particles followed by annealing (61, 62), laser ablation of graphite followed by oxidation and functionalization (63), thermal decomposition of organic compound (64, 65), electrooxidation of graphite (66), and oxidation of candle soot with nitric acid (67). Monodispersed core/shell spinel ferrite/CNPs are formed by thermolysis of metal (Fe^3+^, Co^2+^) oleates followed by carbon coating (68). Highly fluorescent crystalline CNPs have been synthesized by one-step microwave irradiation of sucrose with phosphoric acid at 100 W for 3 min 40 s (69). Activated carbon nanopowder can be produced using a selection of high temperature superfine charcoal via special carbonization, activation, grinding, and classification methods.

The three naturally occurring allotropes of carbon are graphite, diamond, and amorphous carbon. The morphology of CNPs is spherical, and they appear as a black powder. CNPs can be surface functionalized, with organic molecules or polymers chemically bound to the particle surface. Pure carbon has a very low level of toxicity to humans and therefore these nanoparticles can be used. CNPs are being explored widely for use in cancer treatment like breast cancer (70). Studies reveal that cancer treatment using radio waves can heat and destroy a tumor, lymphoma, or metastasized cancer.

### 1.6. Polymer NPs

The NPs are prepared by the polymerization of block copolymers and their self-assembly in solvents into micelles followed by a subsequent stabilization of their structure by core cross-linking. Depending on the type and macrostructure of the block copolymers, the solvent, the concentration, and other process parameters, a variety of core-shell NPs of different shapes (spheres, hollow spheres, ellipsoids, linear and branched strings, etc.) and sizes have been reproducibly synthesized. Most of the NPs are composed of a solid, highly cross-linked core and an elastomeric shell structure (71). Basic spherical or string type NPs can be used as templates for the design of composite structures comprising the basic polymeric NPs and smaller organic, inorganic, or metallic substructures embedded in and attached to the elastomeric shell molecules.

Based on size, geometry, and chemistry various kinds of polymer NPs have a range of utility as adhesives, coating material or impact modifier in medical diagnostics, drug delivery, etc. They can be magnetic particles, electrically conductive particles, or stimuli responsive particles. There are also several classes of biopolymers; for example, nucleic acids—DNA/RNA, fibrous protein, globular structural materials for animals, unbranched polysachharides, lipids, and major structural materials for plants/animals or insects.

Polymer NPs are ideal candidates as drug and gene carriers (72, 73) for various purposes like acne treatment, targeted drug delivery, contraception (Fig. 3), etc. Efficient and targeted delivery of immune-modulatory and immune-stimulatory molecules to appropriate cells is key to the successful development of next-generation vaccines. Polymer-based particulate carriers have emerged as an attractive means for enhancing the delivery efficacy and potency of vaccines and associated immunomodulatory molecules (70, 74, 75).

### 1.7. Quantum dots

Several routes have been used to synthesize quantum dots (QDs) (76) but, generally, techniques for QD synthesis use top–down processing methods and a bottom–up approach. Top–down processing methods include molecular beam epitaxy (MBE), ion implantation, e-beam lithography, and X-ray lithography. Using the alternative bottom–up approach, colloidal QDs are prepared by self-assembly in the solution following a chemical reduction (77–80).

Due to the small structures of QDs, some physical properties such as optical and electron transport characteristics are quite different from those of the bulk materials. QDs, often described as ‘artificial atoms’, exhibit discrete energy levels, and their band gap can be precisely modulated by varying the size (81). QDs are nanometer-scale semiconductor crystals composed of groups II–VI or III–V elements and are defined as particles with physical dimensions smaller than the exciton Bohr radius (82). They exhibit unique luminescence characteristics and electronic properties such as wide and continuous absorption spectra, narrow emission spectra, and high light stability (83).

**Fig. 4:**
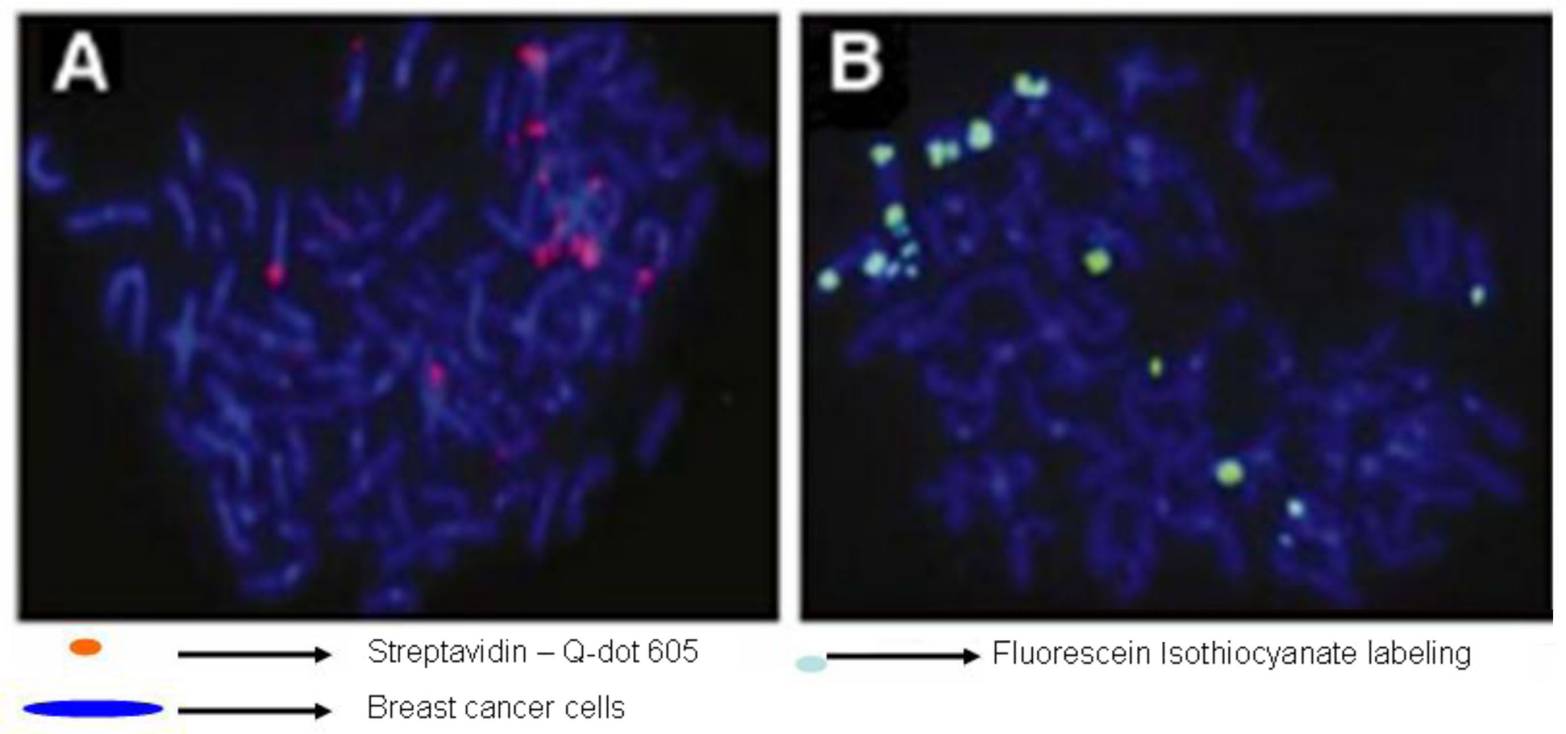
Qualitative FISH detection of HER2 gene-amplified SK-BR3 breast cancer cells with (A) Streptavidin conjugated Qdot 605 and (B) fluorescein isothiocyanate (FITC), respectively. Reproduced from Valizadeh et al., 2012 (83).

Because QDs have constant and unique optical properties, they are the best candidates for cell labeling, as compared with organic dyes. With the application of QDs, single particle tracking (SPT) has the potential to enter into a new era of high resolution and long timescale imaging (84–86). SPT techniques allow scientists to follow single molecules in real time and visualize the actual molecular dynamics in their habitant environment. Using QDs conjugated to anti-M-cadherin antibody, Ishido and Kasuga (87) attempted the visualization of satellite cells in both intact and injured skeletal muscles of rat *in situ*. They demonstrated *in situ* real-time imaging of satellite cells localized within the skeletal muscle. The development of multifunctional nanomaterials combining diagnostic and therapeutic purpose has recently attracted intensive interest (88–93) that includes: 1) biomarker detection in various cancers, 2) imaging and sensing of infectious diseases, and 3) other clinical therapeutic applications. Fig. 4 illustrates qualitative FISH detection of HER2 gene-amplified SK-BR-3 breast cancer cells with streptavidin-conjugated Qdot605 and FITC, respectively. However, the lack of an ideal QD with all positive optical properties and a standard toxicology protocol make it difficult to address the toxicity issue associated with Qdots that is mostly related to leakage of constituent elements, the generation of reactive oxygen species and the environment (94).

### 1.8. Dendrimer

One of the very first dendrimers, the Newkome dendrimers also known as arborol, was synthesized in 1985 (95, 96). Dendrimers can be considered to have three major portions: a core, an inner shell, and an outer shell. Ideally, a dendrimer can be synthesized to have different functionality in each of these portions to control properties such as solubility, thermal stability, and attachment of compounds for particular applications. Synthetic processes can also precisely control the size and number of branches on the dendrimer. There are two defined methods of dendrimer synthesis, divergent synthesis and convergent synthesis. However, the need to protect the active site makes dendrimer synthesis very difficult.

Dendritic molecules are characterized by structural perfection. Dendrimers and dendrons are monodisperse and usually highly symmetric, spherical compounds. The properties of dendrimers are dominated by the functional groups on the molecular surface; however, there are examples of dendrimers with internal functionality (97–99). Also, it is possible to make dendrimers water soluble, unlike most polymers, by functionalizing their outer shell with charged species or other hydrophilic groups. Other controllable properties of dendrimers include toxicity, crystallinity, tecto-dendrimer formation, and chirality (100).

**Fig. 5:**
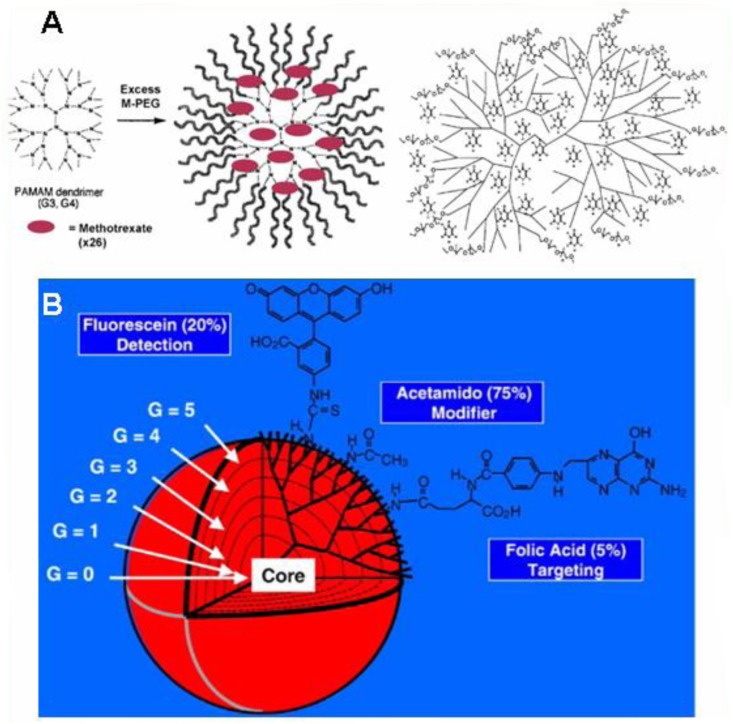
(A) Schematic presentation of the encapsulation of anticancer drugs methotraxate (left) and 5-fluorouracil (right) into PEGylated generation 3 and 4 PAMAM dendrimers and (B) schematic presentation of dendrimers as nano-scaffold for the attachment of cell-specific ligands, modifiers, and fluorescence tags. Reproduced from Svenson and Tomalia, 2012 (104).

Applications of dendrimers typically involves conjugating other chemical species to the dendrimer surface that can function as detecting agents (such as a dye molecule), affinity ligands, targeting components, radioligands, imaging agents, or pharmaceutically active compounds (Fig. 5). Dendrimers have very strong potential for these applications because their structure can lead to multivalent systems. Although there is widespread concern as to the safety of dendrimers, preclinical and clinical experience gained during the development of polymeric excipients, biomedical polymers and polymer therapeutics shows that judicious development of dendrimer chemistry for each specific application will ensure the development of safe and important materials for biomedical and pharmaceutical use.

Carboxylic acid and phenol terminated water-soluble dendrimers were synthesized to establish their utility in drug delivery as well as conducting chemical reactions in their interiors (102). This might allow researchers to attach both targeting molecules and drug molecules to the same dendrimer, which could reduce negative side effects of medications on healthy cells^.^ Globally, dendrimer labs are persistently trying to manipulate dendrimer’s solubilizing trait, in their way to explore dendrimer as drug delivery (101, 103, 104), gene delivery and target specific carrier (105, 106). Scientists have also studied dendrimers for use in sensor technologies. Dendrimers are also being investigated for use as blood substitutes. Their steric bulk surrounding a heme-mimetic center significantly slows degradation compared to free heme, and prevents the cytotoxicity exhibited by free heme (107).

## 2. Potential role of nano-biotechnology in reproductive healthcare

**Fig. 6:**
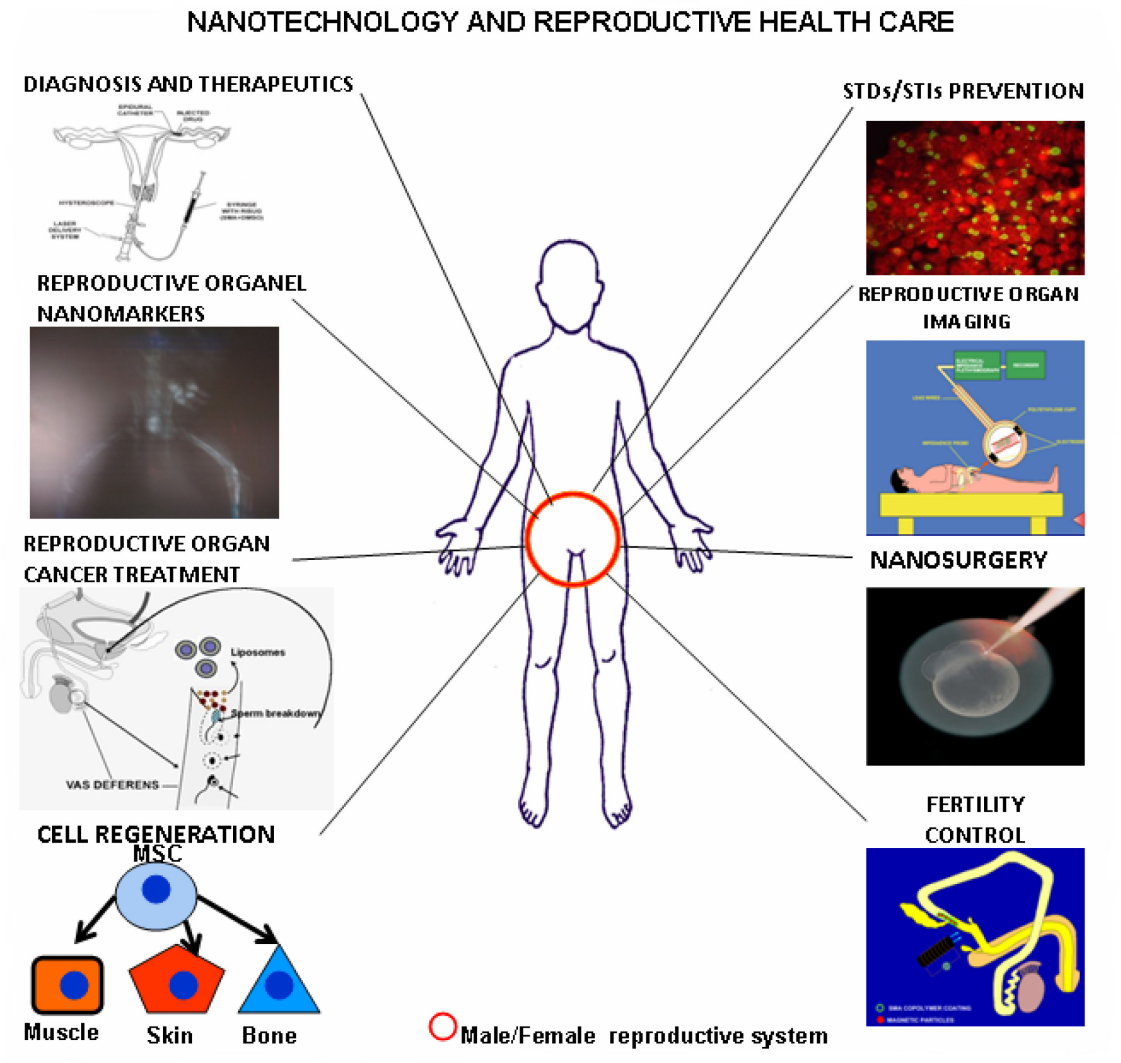
A diagram depicting major possible roles of nano-biotechnology in reproductive healthcare.

Nanoscience and nano-biotechnology is an interdisciplinary field having inputs from various fields like biology, chemistry, physics, mathematics, electronics, etc. Similarly, it has applications in almost all areas of life. Very few people know that this new branch of science has vast potential in the field of reproductive healthcare, one of the most vital domains of medical science and our health (Fig. 6).

### 2.1. Reproductive disease diagnosis and therapeutics

In today’s world, many reproductive diseases go undiagnosed or misdiagnosed, leading to even more complications. Nanotechnology may improve the sensitivity, selectivity, speed, cost, and convenience of diagnosis (108). Nanoscale labeling agents, such as QDs, have numerous advantages to intracellular labeling and visualization. These techniques and others can be further developed to enable enhanced visualization of an array, cell culture, or tumor; be multiplexed to create smaller, denser gene and protein chips; or be integrated into a handheld nanofluidic device to improve clinical diagnosis of a reproductive disease (109).

Nanomarkers give optical contrast and molecular specificity to optical imaging of reproductive organelles and help detect cell organelles as well as suborganelles. Solution biomarkers are an important form of nanotechnology that is able to attach itself to various diseased cells inside the reproductive system enabling to analyze it and treat the person accordingly (110). Some of broadly used nanomarkers for single molecule detection are organic or protein-based fluorescent molecules, luminescent semiconductor NPs, metal NPs, nano diamonds with colored centers, rare earth doped NPs, single walled carbon nanotubes (SWCNTs) (111).

Individual biomolecular interactions can be detected by the deflection of a microcantilever, the red-shifted emission of AuNP, or the altered conductance of a nanowire. Recent studies showed that DNA-conjugated AuNPs are highly promising nanoprobes for the sensitive detection of various biomolecules based on the unique optical and electronic properties (112). Scientists at the California Nanosystems Institute are investigating technology, which increases hormonal detection sensitivity beyond that of traditional sensors, and involves the use of nanoelectronic technologies with the aim of developing a consumer-based, user-friendly sensor for detecting estrogen and progesterone hormone levels (113). Current trials indicate toxic influence of ligand-free AuNP on the fertilizing capability of spermatozoa probably due to remnants of the reducing or stabilizing agents used during AuNP production and not to the NPs themselves (114).

Besides having a role in reproductive organ disease diagnosis, this would also help women in qualifying undesired menopausal symptoms and inform couples seeking infertility treatments on exact timing of conception to occur themselves thus providing a cheaper and faster alternative to current infertility treatments. The sensor measures hormone concentrations using specially made hormone tabs (similar to glucose tabs used by diabetics) made by low cost and precise ink-jet printing of CNTs.

### 2.2. Viral protection and increasing immunity

Similarly, nanotechnology can help in solving significant reproductive healthcare problems (115) like sexually transmitted diseases (STDs) and sexually transmitted infections (STIs). For reproductive healthcare maintenance, contraception, infertility treatment, etc., many people have to undergo surgical intervention like tubectomy, vasectomy or laparoscopy at various stages of life. In order to lessen our pain in future, one will be able to heal wounds a lot faster with the help of new nanotechnological delivery systems that will be sown into bandages and will contain drugs like aluminosilicate, to promote fast regeneration capabilities and it will allow wounds to heal faster (116). Nanotechnology will be able to make this all go a lot faster because, being so tiny, one can theoretically load thousands of nanomechanical device called nanites or nanoids or nanorobots with thousands of different vaccines and inject them into the host all at once and see if any of them work.

As mentioned earlier, RISUG coated intra uterine devices (IUDs) are being developed at IIT Kharagpur that along with giving a contraceptive effect will also have an antimicrobial effect, as successfully tested on female goats, thus helping to avoid infections generated after IUD implantation in women. A new version of RISUG is also being developed to act against human immunodeficiency virus (HIV) that is ready for clinical trials. Antimicrobial effects of silver ion or salts are well known, and although its effect on microorganisms is not clearly explored (117), Ag NPs appears to be the ideal candidate to act against STDs.

### 2.3. Reproductive organ/cell imaging

Imaging is crucial for *in vivo* characterization of the complex behaviors of reproductive disease in time and space that tells us: where it is, how big it is, how fast it is developing, how many molecular processes are contributing simultaneously, what to treat it with, how it is responding to therapy, and how it is changing (118). Because molecules themselves are obviously too small to be imaged directly with non-invasive techniques, the contrast agent should manifest high affinity and avidity for the target organ like reproductive organ. And, unlike traditional blood pool contrast agents, a reproductive site-targeted agent is intended to enhance a selected biomarker that otherwise might be impossible to distinguish from surrounding normal tissue. Molecular imaging actually has been a clinical reality for some time with the use of targeted radionuclides (119).

However, the explosive growth of biocompatible nanotechnologies now promises to expand the horizon for molecular imaging and therapy with a host of novel agents. The desired properties of such targeted contrast agents are: long circulating half-life (hours), selective binding to epitopes of interest, low background signal and prominent contrast-to-noise enhancement, acceptable toxicity profile, ease of production and clinical use, applicability with standard commercially available imaging modalities, and promise for adjunctive therapeutic delivery (120). Clinical availability of these agents is expected to redefine the practice of imaging by focusing on cellular and molecular mechanisms of disease, which will create opportunities for more precise and rational design of conjunctive drug and gene delivery nanosystems.

The contrast mechanism will depend on the choice of imaging modality, which itself is determined by the clinical problem and accessibility for imaging. For example, carrier moieties such as NPs (liposomes or emulsions), dendrimers, viral constructs, buckyballs, or various polymers can be loaded with large payloads of imaging agents such as paramagnetic or superparamagnetic metals, optically active compounds (e.g. fluorescent molecules), or radionuclides to enable detection with standard imaging equipment. In the case of ultrasound imaging, the intrinsic physical properties of the carrier agents themselves (density and compressibility) establish the means for detection (121).

Targeted perfluorocarbon NPs were the first reported molecular imaging agents for ultrasound applications and were shown to augment reflectivity from fibrin thrombi *in vivo* by two orders of magnitude or more (122). Reflective liposomes have also been used to specifically target endothelial integrins that may have huge applications in female reproductive healthcare. ‘Susceptibility’ or ‘cold spot’ imaging agents have been produced by combinations of carriers with iron oxides (e.g. ultra-small particles of iron oxide) or alternative lanthanide species.

QDs NPs have potential use for non-invasive investigation of mammalian spermatozoa (123). Research has shown that BRET-QD conjugated with boar spermatozoa helped to understand the sperm behavior inside the uterus followed by their impact on sperm motility, viability and fertilizing potential (124). QD-based near-infrared (NIR) fluorescence cancer imaging is a growing field for both preclinical and clinical application to the clinical management for cancer patients due to its advantageous features, including a high spatial resolution, portability, real-time display and detailed molecular profiling with the multiplexed use of fluorescent probes and therefore it can play vital role in imaging reproductive organ cancer (125).

### 2.4. Reproductive organ cancer treatment

NPs like CNPs and a range of nanodevices like fullerenes are giving us immense hope against reproductive organ cancer. For instance, nanoshells work similarly to NPs but instead of injecting the cancer cells with chemotherapy (126), they will simply use the heat from infrared light. It has been discovered that when nanites are irradiated with X-rays, the nanites produce their own electrons that can be used to target cancer cells and destroy them without harming the rest of the body. Nanites can also scatter through the body to detect cancer cells and tag them so that doctors know exactly where in the body that cancer has spread to. That in turn will help physicians to avoid chemotherapy for example, Qdots the gold nanites are able to track down cancer cells in the body.

NPs will be able to inject chemotherapy directly into cancer cells themselves with very minimal damage to the surrounding cells (127). CNPs have shown immense potential against breast cancer. Metallic nanoparticles like gold (AuNPs) hold great promise as PDT and hyperthermic agents. Upon X-ray irradiation, AuNP can induce cellular apoptosis through the generation of radicals. This may have tremendous potential to kill ovarian cancer cells without harming the surrounding healthy tissue (55–57). NPs can also act as drug delivery agents, for example, hypericin-loaded NPs (125) for the photodynamic treatment of cancer. Drug delivery with CNTs for *in vivo* treatment of reproductive organ cancer like uterine cancer, cervical cancer, vaginal and vulvar cancer in females and prostate cancer or penile cancer in males is another possibility. RISUGadv invented by Prof. Sujoy K. Guha prevents prostate cancer, the most prevalent cancer in elderly man. Also, RISUG-PH studies in small animals have the potential to act against benign prostate hyperplasia (BPH).

### 2.5. Nano surgery

Nanorobots can play a significant role in laparoscopic reproductive organ surgery and to correct abnormalities. Surgical nanorobots could be introduced into the body through the vascular system or at the ends of catheters into various vessels and other cavities in the human body. A nanorobot programmed by a surgeon could act as a semi-autonomous on-site surgeon inside the body. Such a device could perform various functions like searching for pathology in reproductive as well as other organs, and then diagnosing and correcting lesions or cysts by nanomanipulation, coordinated by a computer thus maintaining contact with the supervising surgeon via coded ultrasound signals (128).

The earliest forms of cellular nanosurgery have already been explored. For example, a rapidly vibrating (100 Hz) micropipette with a <1 nm tip diameter has been used to cut dendrites from single neurons without damaging cell viability. Axotomy of roundworm neuron was performed by femtosecond laser surgery after which the axons functionally regenerated. A femtolaser acts like a pair of nano-scissors by vaporizing tissue locally while leaving adjacent tissue unharmed. The procedure does not kill the cell on which nanosurgery was performed (129). Atomic force microscopes have also been used for the dissection of a bacterium cell wall *in situ* in aqueous solution, with 26 nm thick twisted strands revealed inside the cell wall after mechanically peeling back large patches of the cell wall.

### 2.6. Cell regeneration

Since natural tissues or organs are in nanometer dimension and cells directly interact with (and create) nanostructured extra-cellular matrices (ECM), the biomimetic features and excellent physiochemical properties of nanomaterials play a key role in stimulating cell growth as well as to guide tissue regeneration. Even though it was a field in its infancy a decade ago, numerous researchers are currently fabricating cytocompatible biomimetic nanomaterial scaffolds encapsulating cells (such as stem cells, chondrocytes and osteoblasts, etc.) for tissue engineering applications. Nanomaterials exhibit superior cytocompatible, mechanical, electrical, optical, catalytic and magnetic properties compared to conventional microsized materials. These unique properties help to improve various tissue growth over what is achievable today (130).

The stem-cell-technology has a new role to play in reproduction. Firstly, the stem-cell source could be pooled out of slaughter-house oocytes or from the vast pool of embryos hatching out in many animal species. These cells can transform themselves into 200 or more cell types, which could be used to repair or regenerate new desired cells. This advanced cell research may help people suffering from reproductive organ cancer or persons devoid of genital organs (131). Nanobots are 2.5 times smaller than DNA that can enter individual cells and repair them. With that concept, nanotechnology will be able to cure just about everything because all problems start at a cellular level (132).

### 2.7. Contraception and infertility management

The use of contraceptives should not only prevent pregnancy but also help the individual to maintain good health. A research team at the Indian Institute of Technology Kharagpur has been working on several polymer-based fertility control molecules (13–17) that owe a lot to nanotechnology. The Cuproferrogel nanomedicine iron oxide–copper–styrene maleic anhydride–dimethyl sulfoxide called Smart RISUG (Figure 3) developed in our laboratory has proved to be very effective against sperm as well as ovum, enables controlled delivery to the target site which in this case is vas deferens/fallopian tube, controlled distribution with the help of external electromagnetic field and most importantly non-invasive imaging by X-ray computer tomography (CAT) scan, MRI, electrical impedance plethysmography, etc. (13–15, 110, 118, 126). Similarly, we are working on an antimicrobial vaginal contraceptive.

There are so many oral and injectable short-term contraceptives available over the counter throughout the world. Oral contraception is preferred in the western world while IUDs are mostly used in developing countries due to a lack of reliable alternatives in market beside permanent sterilization like vasectomy and female sterilization that itself is invasive and associated with several side-effects and complications. Nanotechnology can improve the dose required, efficacy, and delectability of many fertility control agents. Electromechanical devices and radiothermy may also help in proper biodistribution of the fertility control agent (15) and also reversibility when desired by the couple. For the pharmaceutical industries novel drug delivery technologies (133) can address issues associated with current pharmaceutics such as extending product life, enhancing their performance and acceptability either by increasing efficacy or improving safety and product compliance.

Many people are not capable of reproducing because their bodies are not good hosts to a desirable environment. With the aid of nanotechnology, these little nanobots may quickly go to work at reconstructing genitals and other reproductive features so that one can once reproduce. However, it is easier said than done to treat infertility problems with such ease as a major portion of infertility cases are unexplained. But at least we can approach the problem in a better way with the help of advanced nanotechnological tools and monitoring devices. Sensors discussed in section 3.1 or ‘fertility chip’ have huge potential as a treatment for male fertility in the short-term (134) and female in the long-term. Additionally, as the chip is of nano-proportions, the patient will have minimum discomfort while the information generated will be invaluable for prospective patients.

## 3. Future perspectives of nanotechnology for reproductive health

On one hand, the exploding population in developing countries like India and China is a major issue affecting our socio-economic development; and the other hand reduced fertility is a sensitive problem emerging globally due to lifestyle changes and environmental factors (135). Good reproductive health in turn effects our socio-economic development directly or indirectly by eradicating poverty, to achieve primary education when family size is small, promote gender equality and empower women, reduce child mortality; improve maternal health, combat HIV/AIDS/STDs/STIs, etc., ensuring environmental sustainability and global partnerships in a bigger scenario (136).

The potential of nanotechnology offers some exciting possibilities in reproductive healthcare. Some techniques are only imagined, while others are at various stages of testing, or actually being used today. The use of nanotechnology in the field of reproductive biomedicine can revolutionize the way we detect and treat damage to the human body and disease, and many techniques only imagined a few years ago are making remarkable progress towards becoming realities. For instance, this paper has described a range of antimicrobial, long-term, stable, single-use male and female injectable contraceptives being developed like RISUG in advanced phase III clinical trials (137, 138); and its nanotechnological versions like Smart RISUG (13–17).

Other reproductive biomedicines are being developed at IIT Kharagpur to prevent cancer; for example, Invivgensome (liposome synthesized inside the testis) invented by Prof. Guha prevents prostate cancer development which is one of most prevalent cancers in elderly males, and RISUG-PH acts against BPH for which trials are about to begin. Liposomes have great reproductive healthcare potential both when developed *in vitro* and also when self-generated *in vivo*. Another nanotechnology-based tool for reproductive organ cancer treatment is fullerenes described as a nanoscale molecule that is made up of only carbon. Carbon, as we know, is the basis of nature’s construction and, therefore, also represents our very own construction. Fullerenes (139) allow us to build nanostructures, so that one may integrate our own programming and machinery that will go on to perform marvelous tasks *in situ*.

With respect to STDs or STIs, RISUG has also shown primary evidence to work against HIV, and RISUG-coated IUDs do not allow microbes in its vicinity when placed inside the body after child birth (140). Although nanotechnology does not appear to play a direct role in infertility treatment, indirectly it can play an immense role by helping in early, low-cost and accurate detection of disease sites with smart sensors, detection of hormone levels and non-invasive imaging of nanomedicines placed in the reproductive tube.

The reasons behind most deaths today are either late diagnosis, inability to diagnose the main reason or location of disease or misinterpretation of data. We do hope nanotechnology, for instance nanites, will one day be able to scurry throughout our bodies via the circulatory system (traveling through our blood) and monitor every single vital sign that exists (141), for example, whether there are any broken bones, torn muscle tissue, irregularities, screen metabolism levels, observe cholesterol levels, monitor hormone levels, make sure that the organs are functioning properly, and any other requirement for a healthy body.

Some companies are developing nanotubes to heal broken bones by providing bones with a proper structure in order for them to grow back in the way that they are supposed to (142). CNTs are still a relatively unexplored area in a rapidly advancing field. Any amount of improvements can be made to CNTs through various techniques (143) because of their great material properties. For example, it was shown that by electrospinning and plasma-functionalizing SWCNTs, adhesion to surrounding polymer matrices was greatly improved along with the tensile properties of the nanotubes. Also, we know that most nanotubes are cleared from the body very quickly after being distributed throughout (144). This decreases the chances of higher toxicity levels in the blood. The good functionalization of SWCNs allows us to attach a number of groups to the tubes for different systems. Radioactive labels could be attached for use in reproductive organ bioimaging (145). It was shown that CNTs were used to efficiently deliver drugs to specific cancer cells of the epithelium (146).

Nanotechnology may also be able to aid and even perfect the act of regenerating cells/tissues (130). Regeneration is the process of bringing a person back to life. Today, there are many different problems with doing so but nanotechnology may be able to fix most if not all of them. One of the biggest problems is due to the crystallization of frozen cells but nanotechnology may be able to warm those cells and even remake some of them so that the person doesn’t biologically fall apart when they’re revived. Nanotechnology may be able to also simply cure cell damage as soon as we die which means we wouldn’t even have to be frozen first.

As discussed previously, most of our nanotechnology-based future healthcare expectations are based on molecular nanotechnology (MNT). MNT is a technology based on the ability to build structures to complex, atomic specifications by means of mechanosynthesis (147). This is distinct from nanoscale materials. Based on Richard Feynman’s vision of miniature factories using nanomachines to build complex products (including additional nanomachines), this advanced form of nanotechnology (or molecular manufacturing) would make use of positionally controlled mechanosynthesis guided by molecular machine systems. MNT would involve combining physical principles demonstrated by chemistry, other nanotechnologies, and the molecular machinery of life with the systems engineering principles found in modern macroscale factories (148).

Nanotechnology in medicine called nanomedicine involves applications of NPs currently under development, as well as longer ranges research that involves the use of manufactured nano-robots to make repairs at the cellular level (149). Future applications of nanomedicine will be based on the ability to build nanorobots. These nanorobots will actually be programmed to repair specific diseased cells, functioning in a similar way to antibodies in our natural healing processes. This way, nanomedicine offers great promise for the future, especially the mixing of diagnostic and therapeutic capabilities in healthcare. Nano surgery like laparoscopy is already being used for reproductive health problem detection, corrective surgery, tubal sterilization, chronic pelvic pain, etc. since past many years (150).

Future nanorobots equipped with operating instruments and mobility will be able to perform precise and refined intracellular surgeries in reproductive organs, which are beyond the capabilities of direct manipulation by the human hand. We envision biocompatible surgical nanorobots that can find and eliminate isolated cancerous reproductive cells, remove microvascular obstructions and recondition vascular endothelial cells, perform non-invasive tissue and organ transplant, conduct molecular repairs on traumatized extracellular and intracellular structures, and even exchange new complete chromosomes for old ones inside individual living human cells (128).

The future of nanomedicines is undermined by the lack of financial profitability, consumer distrust, and ineffective regulation of new and generic products, weak patent protection and insurance market failure. Its economic breakthrough is dependent on a series of countervailing measures and actions. Success requires more investment induced by cost–effectiveness analyses and business plans based on clinical data, public education based on nanotoxicology studies, smart regulatory reform in the areas of testing, market entry and liability, effective and strategic patenting, patent dispute prevention and resolution, and innovative insurance policies.

## References

[1] Allhoff F (2009). The coming era of nano medicine. Am J Bioeth.

[2] EU Technology Platform on Nanomedicine http://www.etp-nanomedicine.eu/public.

[3] Emerich DF, Halberstadt C, Thanos C (2007). Role of nano-biotechnology in cell-based nanomedicine: a concise review. J Biomed Nanotechnol.

[4] Kedziora A, Gorzel Anczyk K, Ploskonska GB (2013). Positive and negative aspects of silver nanoparticles usage. Biol Int.

[5] Buzea C, Blandino IIP, Robbie K (2007). Nanomaterials and nanoparticles: sources and toxicity. Biointerphases.

[6] Alivisatos P (2004). The use of nanocrystals in biological detection. Nat Biotechnol.

[7] Madani SY, Mandel A, Seifalian AM (2013). A concise review of carbon nanotube’s toxicology. Nano Rev.

[8] Baughman RH, Anvar AZ, Walt AH (2002). Carbon nanotubes – the route toward applications. Science.

[9] Ciofani G, Raffa V, Menciassi A, Cuschieri A (2009). Boron nitride nanotubes: an innovative tool for nanomedicine. Nano Today.

[10] Bonnemain B (1998). Superparamagnetic agents in magnetic resonance imaging, physicochemical characteristics and clinical applications a review. J Drug Target.

[11] United Nations (1995). Population and Development, Vol. 1: Programme of Action adopted at the International Conference on Population and Development: Cairo, 5-13 September 1994, paragraph 7.2.

[12] United Nations (2002). World Youth Report 2003: Report of the Secretary- General (E/CN.5/2003/4), para. 16.

[13] Jha RK, Jha PK, Guha SK (2009a). Smart RISUG: a potential new contraceptive and its magnetic field mediated sperm interaction. Int J Nanomed.

[14] Jha RK, Jha PK, Rana SVS, Guha SK (2009b). Spermicidal action of styrene maleic anhydride polyelectrolyte in combination with magnetic and electrically conductive particles. Int J Pharmacol.

[15] Jha R, Jha PK, Rana SVS, Guha SK (2010a). An approach to non-invasive delivery, biodistribution and fertility control potential evaluation of Cuproferrogel Fe3O4–Cu–SMA–DMSO in female. Fertil Steril.

[16] Jha PK, Jha R, Gupta BL, Guha SK (2010b). Effect of γ-dose rate and total dose interrelation on the polymeric hydrogel: a novel injectable male contraceptive. Radiat Phys Chem.

[17] Jha PK, Jha R, Datt R, Guha SK (2011). Entropy in good manufacturing practices: a tool for quality assurance. Eur J Oper Res.

[18] Na HB, Song IC, Hyeon T (2009). Inorganic nanoparticles for MRI contrast agents. Adv Mater.

[19] Gil PR, Huhn D, Mercato LL, Sasse D, Parak WJ (2010). Nanopharmacy: inorganic nanoscale devices as vectors and active compounds. Pharmacol Res.

[20] Rube MA, Cox BF, Gueorguieva M, Kakchingtabam D, Andre P, Mezler A (2013). Iron_platinum alloy nanoparticles for guidewire and resonant markers for catheter localization during interventional MRI. Biomed Engin/Biomed Tech.

[21] Radwan SH, Azzazy HME (2009). Gold nanoparticles for molecular diagnostics. Expert Rev Mol Diagn.

[22] Spivak MY, Bubnov RV, Yemets IM, Lazarenko LM, Tymoshok NO, Ulberg ZR (2013). Gold nanoparticles – the theranostic challenge for PPPM: nanocardiology application. EPMA J..

[23] Bawa R (2008). Nanoparticle based therapeutics in human: a survey. Nanotechnol Law Bus.

[24] Qin W, Din D, Liu J, Yuan WZ, Hu Y, Liu B (2012). Biocompatible nanoparticles with aggregation-induced emission characteristics as far-red/near-infrared fluorescent bioprobes for in vitro and in vivo imaging applications. Adv Funct Mater.

[25] Schwartz JA, Shetty AM, Price RE, Stafford RJ, Wagon JC, Uthamanthil RK (2008). Feasibility study of particle-assisted laser ablation of brain tumors in orthotopic canine model. Cancer Res.

[26] Wang Y, Chen L (2011). Quantum dots lighting up the research and development of nanomedicine. Nanomedicine.

[27] Wagh A, Qian SY, Law B (2012). Development of biocompatible polymeric nanoparticles for in vivo NIR and FRET imaging. Bioconjugate Chem.

[28] Tang R, Lee H, Achilefu S (2012). Induction of pH sensitivity on the fluorescence lifetime of quantum dots by NIR fluorescent dyes. Am Chem Soc.

[29] Chuah AM, Kuroiwa T, Ichikawa S, Kobayashi I, Nakajima M (2009). Formation of biocompatible nanoparticles via the self-assembly of Chitosan and Modified Lecithin. J Food Sci.

[30] Sanna V, Pintus G, Roggio AM, Punzoni S, Posadino AM, Arca A (2011). Targeted biocompatible nanoparticles for the delivery of (-)-epigallocatechin 3-gallate to prostate cancer cells. J Med Chem.

[31] Barandeh F, Nguyen PL, Kumar R, Lacobucci GJ, Kuznicki ML, Kosterman A (2012). Organically modified silica nanoparticles are biocompatible and can be targeted to neurons in vivo. PLoS One.

[32] Aswathy RG, Sivakumar B, Brahatheeswaran D, Fukuda T, Yoshida Y, Maekawa T (2012). Biocompatible fluorescent zein nanoparticles for simultaneous bioimaging and drug delivery application. Adv Nat Sci: Nanosci Nanotechnol.

[33] Roullin VG, Callewaert M, Delavoie F, Molinari M, Seconde A, Andry MC (2010). Optimised NSAIDs-loaded biocompatible nanoparticles. Nano-Micro Lett.

[34] Chan JM, Zhang L, Tong R, Ghosh D, Gao W, Liao G (2009). Spatiotemporal controlled delivery of nanoparticles to injured vasculature. Proc Natl Acad Sci U S A.

[35] Kamaly N, Xiao Z, Valencia PM, Radovic AF, Farokhzad OC (2012). Targeted polymeric therapeutic nanoparticles: design, development and clinical translation. Chem Soc Rev.

[36] Bangham AD, Horne RW (1962). Action of saponin on biological cell membranes. Nature.

[37] Torchilin V (2006). Multifunctional nanocarriers. Adv Drug Deliv Rev.

[38] Woodle MC, Papahadjopoulos D (1989). Liposome preparation and size characterization. Methods Enzymol.

[39] Bergin C, O’Leary A, McCreary C (1995). Treatment of Kaposi’s sarcoma with liposomal doxorubicin. Am J Health Syst Pharm..

[40] GreGoriadis G (1995). Engineering liposomes for drug delivery: progress and problem. Trends Biotechnol.

[41] Gouin S (2004). Microencapsulation: industrial appraisal of existing technologies and trends. Trends Food Sci Tech.

[42] Qian J, Wang W, Li Y, Xu Y, Sun Q (2012). Optical extinction properties of perforated gold-silica-gold multilayer Nanoshells. J Phys Chem.

[43] Odom TW, Huang JL, Kim P, Lieber CM (1998). Atomic structure and electronic properties of single-walled carbon nanotubes. Nature.

[44] Liu Z, Tabakman S, Welsher K, Dai H (2009). Carbon nanotubes in biology and medicine: in vitro and in vivo detection, imaging and drug delivery. Nano Res.

[45] Martin CR, Kohli P (2003). The emerging field of nanotube biotechnology. Nat Rev Drug Discov.

[46] Klumpp C, Kostarelos K, Prato M, Bianco A (2006). Functionalized carbon nanotubes as emerging nanovectors for the delivery of therapeutics. Biochim Biophys Acta.

[47] Raffa V, Vittorio O, Riggio C, Cuschieri A (2010). Progress in nanotechnology for healthcare. Minim Invasive Ther Allied Tech.

[48] Hersam MC (2008). Progress towards monodisperse single-walled carbon nanotubes. Nat Nanotechnol.

[49] Sun Y, Xia Y (2002). Shape-controlled synthesis of gold and silver nanoparticles. Science.

[50] Liao H, Nehl CL, Hafner JH (2006). Biomedical applications of plasmon resonant metal nanoparticles. Nanomedicine.

[51] Zhang Q, Large N, Nordlander P, Wang H (2014). Porous gold Au with tunable Plasmon resonances and intense field enhancements for single particle SERS. J Phys Chem Lett.

[52] Mukherjee P, Bhattacharya R, Wang P, Wang L, Basu S, Nagy JA (2005). Antiangiogenic properties of gold nanoparticles. Clin Cancer Res.

[53] Mukherjee P, Bhattacharya R, Bone N, Lee YK, Patra CR, Wang S (2007). Potential therapeutic application of gold nanoparticles in B-chronic lymphocytic leukemia (BCLL): enhancing apoptosis. J Nanobiotechnol.

[54] Sigler JW, Bluhm GB, Duncan H, Sharp JT, Ensign DC, Mccrum WR (1974). Gold salts in the treatment of rheumatoid arthritisA double-blind study. Ann Intern Med.

[55] Chatterjee DK, Fong LS, Zhang Y (2008). Nanoparticles in photodynamic therapy: an emerging paradigm. Adv Drug Deliv Rev.

[56] Juzenas P, Chen W, Sun Y, Coelho MANC, Generalov R, Generalova N (2008). Quantum dots and nanoparticles for photodynamic and radiation therapies of cancer. Adv Drug Deliv Rev.

[57] Paszko E, Ehrhardt C, Senge MO, Kelleher DP, Reynolds JV (2011). Nanodrug applications in photodynamic therapy. Photodiagnosis Photodyn Ther.

[58] Gupta AK, Gupta M (2005). Synthesis and surface engineering of iron oxide nanoparticles for biomedical applications. Biomaterials.

[59] Rasmussen JW, Martinez E, Louka P, Wingett DG (2010). Zinc oxide nanoparticles for selective destruction of tumor cells and potential for drug delivery applications. Expert Opin Drug Deliv.

[60] Jeng HA, Swanson J (2006). Toxicity of metal oxide nanoparticles in mammalian cells. J Environ Sci Health A Tox Hazard Subst Environ Eng.

[61] Krasheninnikov AV, Banhart F (2007). Engineering of nanostructured carbon materials with electron or ion beams. Nat Mater.

[62] Ray SC, Saha A, Jana NR, Sarkar R (2009). Fluorescent carbon nanoparticles: synthesis, characterization, and bioimaging application. J Phys Chem C.

[63] Puretzky AA, Geohegan DB, Fan X, Pennycook SJ (2000). Dynamics of single-wall carbon nanotube synthesis by laser vaporization. Appl Phys A.

[64] Nessim GD, Seita M, O’Brien KP, Hart AJ, Bonaparte RK, Mitchell RR (2009). Low temperature synthesis of vertically aligned carbon nanotubes with electrical contact to metallic substrates enabled by thermal decomposition of the carbon feedstock. Nano Lett.

[65] Jin YH, Seo SD, Shim HW, Park KS, Kim DW (2012). Synthesis of core/shell spinel ferrite/carbon nanoparticles with enhanced cycling stability for lithium ion battery anodes. Nanotechnology.

[66] Zhao QL, Zhang ZL, Huang BH, Peng J, Zhang M, Pang DW (2008). Facile preparation of low cytotoxicity fluorescent carbon nanocrystals by electrooxidation of graphite. Chem Commun.

[67] Tian L, Ghosh D, Chen W, Pradhan S, Chang X, Chen S (2009). Nanosized carbon particles from natural gas soot. Chem Mater.

[68] Chen IH, Wang CC, Chen CY (2010). Fabrication and characterization of magnetic cobalt ferrite/polyacrylonitrile and cobalt ferrite/carbon nanofibers by electrospinning. Carbon.

[69] Yang Y, Cui J, Zheng M, Hu C, Tan S, Xiao Y (2012). One-step synthesis of amino-functionalized fluorescent carbon nanoparticles by hydrothermal carbonization of chitosan. Chem Commun.

[70] Yezhelyev MV, Gao X, Xing Y, Al-Hajj A, Nie S (2006). O’Regan RM. Emerging use of nanoparticles in diagnosis and treatment of breast cancer. Lancet Oncol.

[71] Bronich TK, Keifer PA, Shlyakhtenko LS, Kabanov AV (2005). Polymer micelle with cross-linked ionic core. J Am Chem Soc.

[72] Li Y, Gao GH, Lee DS (2013). Stimulus-sensitive polymeric nanoparticles and their applications as drug and gene carriers. Adv Healthc Mater.

[73] Haley B, Frenkel E (2008). Nanoparticles for drug delivery in cancer treatment. Urol Oncol.

[74] Paciotti GF, Myer L, Weinreich D, Goia D, Pavel N, McLaughlin RE (2004). Colloidal gold: a novel nanoparticle vector for tumor directed drug delivery. Drug Deliv.

[75] Leleux J, Roy K (2013). Micro and nanoparticle-based delivery systems for vaccine immunotherapy: an immunological and materials perspective. Adv Healthc Mater.

[76] Bera D, Qian L, Tseng T-K, Holloway PH (2010). Quantum dots and their multimodal applications: a review. Materials.

[77] Mattoussi H, Palui G, Na HB (2012). Luminescent quantum dots as platforms for probing in vitro and in vivo biological processes. Adv Drug Deliv Rev.

[78] Birudavolu S, Nuntawong N, Balakrishnan G, Xin YC, Huang S, Lee SC (2004). Selective area growth of InAs quantum dots formed on a patterned GaAs substrate. Appl Phys Lett.

[79] Nakata Y, Mori T, Seki H (2000). Molecular beam epitaxial growth of InAs self-assembled quantum dots with light-emission at 1.3 µm. J Cryst Growth.

[80] Yamilov A, Herrera MR, Bertino MF (2007). Quantum dots by ultraviolet and x-ray lithography. Nanotechnology.

[81] Klimov VI (2007). Spectral and dynamical properties of multiexcitons in semiconductor nanocrystals. Annu Rev Phys Chem.

[82] Chan WC, Maxwell DJ, Gao X, Bailey RE, Han M (2002). Luminescent quantum dots for multiplexed biological detection and imaging. Curr Opin Biotechnol.

[83] Valizadeh A, Mikaeili H, Samiei M, Farkhani SM, Zarghami N, Akbarzadeh A (2012). Quantum dots: synthesis, bioapplications, and toxicity. Nanoscale Res Lett.

[84] Chang Y-P, Pinaud F, Antelman J, Weiss S (2008). Tracking bio-molecules in live cells using quantum dots. J Biophotonics.

[85] Cherry RJ (1992). Keeping track of cell surface receptor. Trends Cell Biol.

[86] Saxton MJ, Jacobson K (1997). Single-particle tracking: applications to membrane dynamics. Annu Rev Biophys Biomol Struct.

[87] Ishido M, Kasuga N (2011). In situ real-time imaging of the satellite cells in rat intact and injured soleus muscles using quantum dots. Histochem Cell Biol.

[88] Lim IIS (2008). Gold and magnetic oxide/gold core/shell nanoparticles as bio-functional nanoprobes. Nanotechnology.

[89] Duan H, Nie S (2007). Cell-penetrating quantum dots based on multivalent and endosome-disrupting surface coatings. J Am Chem Soc.

[90] Smith AM (2008). Bioconjugated quantum dots for in vivo molecular and cellular imaging. Adv Drug Deliv Rev.

[91] Wang L (2008). Core@shell nanomaterials: gold-coated magnetic oxide nanoparticles. J Mater Chem.

[92] Park K (2009). New generation of multifunctional nanoparticles for cancer imaging and therapy. Adv Funct Mater.

[93] Wu W (2010). In-situ immobilization of quantum dots in polysaccharide-based nanogels for integration of optical pH-sensing, tumor cell imaging, and drug delivery. Biomaterials.

[94] Bottrill M, Green M (2011). Some aspects of quantum dot toxicity. Chem Commun.

[95] Dewald JR, Tomalia DA (1985). Dense star polymers having core, core branches, terminal groups. U.S. Patent 4.

[96] Tomalia DA, Baker H, Dewald J, Hall M, Kallos G, Martin S (1985). “A New Class of Polymers: Starburst-Dendritic Macromolecules”. Polymer Journal.

[97] Antoni P, Hed Y, Nordberg A, Nyström D, von Holst H, Hult A (2009). Bifunctional dendrimers: from robust synthesis and accelerated one-pot postfunctionalization strategy to potential applications. Angew Chem Int Ed Engl.

[98] McElhaonon JR, McGrath DV (2000). Toward chiral polyhydroxylated dendrimers. Preparation and chiroptical properties. J Org Chem.

[99] Liang CO, Frechet JMJ (2005). Incorporation of functional guest molecules into an internally functionalizable dendrimers through olefin metathesis. Macromolecules.

[100] Nanjwade BK, Bechraa HM, Derkara GK, Manvia FV, Nanjwade K (2009). Dendrimers: emerging polymers for drug-delivery systems. Eur J Pharm Sci.

[101] Svenson S, Tomalia DA (2012). Dendrimers in biomedical applications- reflections on the field. Adv Drug Deliv Rev.

[102] Kannumalle LS, Ramesh R, Maddipatla MVSN, Nithyanandan J, Ramamurthy V (2005). Dendrimers as photochemical reaction media. Photochemical behavior of unimolecular and bimolecular reactions in water-soluble dendrimers. J Org Chem.

[103] Khopade AJ, Caruso F, Tripathi P, Nagaich S, Jain NK (2002). “Cascade”- and “Effect of dendrimer on entrapment and release of bioactive from liposomes. Int J Pharm.

[104] Prajapati RN, Tekade RK, Gupta U, Gajbhiye V, Jain NK (2009). Dendimer-mediated solubilization, formulation development and in vitro–in vivo assessment of piroxicam. Synthesis.

[105] Chauhan AS, Sridevi S, Chalasani KB, Jain AK, Jain SK, Jain NK (2003). Dendrimer-mediated transdermal delivery: enhanced bioavailability of indomethacin. Synthesis.

[106] Kukowska-Latallo JF, Candido KA, Cao Z, Nigavekar SS, Majoros IJ, Thomas TP (2005). Nanoparticle targeting of anticancer drug improves therapeutic response in animal model of human epithelial. Synthesis.

[107] Twyman LJ, Ellis A, Gittins PJ (2012). Pyridine encapsulated hyperbranched polymers as mimetic models of haeme containing proteins, that also provide interesting and unusual porphyrin–ligand geometries. Chem Commun.

[108] Yager P, Edwards T, Fu E, Helton K, Nelson K, Tam MR (2006). Microfluidic diagnostic technologies for global public health. Nature.

[109] Lee WG, Kim YG, Chung BG, Demirci U, Khademhosseini A (2010). Nano/Microfluidics for diagnosis of infectious diseases in developing countries. Adv Drug Deliv Rev.

[110] Horne AW, Duncan WC, Critchley HO (2010). The need for serum biomarker development for diagnosing and excluding tubal ectopic pregnancy. Acta Obstet Gynecol Scand.

[111] Bates M, Huang B, Dempsey GT, Zhuang X (2007). Multicolour super-resolution imaging with photo switchable fluorescent probes. Science.

[112] Wang L, Song S, Pan D, Li D, Fan C (2010). Gold nanoparticles based sensing strategies for biomolecular detection. Pure App Chem.

[113] Hennequin Y, Allier CP, McLeod E, Mudanyali O, Migliozzi D, Ozcan A (2013). Optical detection and sizing of single nanoparticles using continuous wetting films. ACS Nano.

[114] Barchanski A, Taylor U, Klein S, Petersen S, Rath D, Barcikowski S (2011). Golden perspective: application of laser-generated gold nanoparticle conjugates in reproductive biology. Reprod Domest Anim.

[115] Betty YSK, Rutka JT, Chan WCW (2010). Current concepts. Nanomedicine. N Eng J Med.

[116] Chu Y, Yu D, Wang P, Xu J, Li D, Ding M (2010). Nanotechnology promotes the full-thickness diabetic wound healing effect of recombinant human epidermal growth factor in diabetic rats. Wound Rep Reg.

[117] Kim JS, Kuk E, Yu KN, Kim JH, Park SJ, Lee HJ (2007). Antimicrobial effects of silver nanoparticles. Nanomedicine.

[118] Wickline SA, Lanza GM (2003). Nanotechnology for molecular imaging and targeted therapy. Circulation.

[119] Ersahin D, Doddamane I, Cheng D (2011). Targeted radionuclide therapy. Cancers.

[120] Choi HS, Frangioni JV (2010). Nanoparticles for biomedical imaging: fundamentals for clinical translation. Mol Imaging.

[121] Thomson K, Varma D (2010). Safe use of radiographic contrast media. Australian Prescriber.

[122] Frangioni JV, Hajjar RJ (2004). *In vivo* tracking of stem cells for clinical trials in cardiovascular disease. Circulation.

[123] Feugang JM, Youngblood RC, Greene JM, Fahad AS, Monroe WA, Willard ST (2012). Application of quantum dot nanoparticles for potential non-invasive bio-imaging of mammalian spermatozoa. J Nanobiotechnol.

[124] So MK, Loening AM, Gambhir SS, Rao J (2006). Creating self-illuminating quantum dot conjugates. Nat Protoc.

[125] Kosaka N, Ogawa M, Choyke PL, Kobayashi H (2009). Clinical implications of near-infrared fluorescence imaging in cancer. Future Oncol.

[126] Bardhan R, Lal S, Joshi A, Halas NJ (2011). Theranostic nanoshells: from probe design to imaging and treatment of cancer. Accounts Chem Res.

[127] Liang XJ, Chen C, Zhao Y, Wang PC (2010). Circumventing tumor resistance to chemotherapy by nanotechnology. Methods Mol Biol.

[128] Cavalcanti A, Shirinzadeh B, Murphy D, Smith JA (2007). Nanorobots for laparoscopic cancer surgery. 6th IEEE/ACIS International Conference on Computer and Information Science.

[129] Fatih M (2010). Nerve regeneration in *Caenorhabditis elegans* after femtosecond laser axotomy.

[130] Zhang L, Webster TJ (2009). Nanotechnology and nanomaterials: promises for improved tissue regeneration. Nano Today.

[131] Calin M, Stan D, Simion V (2013). Stem cell regenerative potential combined with nanotechnology and tissue engineering for myocardial regeneration. Curr Stem Cell Res Ther.

[132] Loizidou M, Seifalian AM (2010). Nanotechnology and its applications in surgery. Br J Surg.

[133] Sahoo SK, Labhasetwar V (2003). Nanotech approached to drug delivery and imaging. Drug Discov Today.

[134] Rae MT, Price D, Harlow CR, Critchley HOD (2009). Glucocorticoid receptor-mediated regulation of MMP9 gene expression in human ovarian surface epithelial cells. Fertil Steril.

[135] Project Report No. 2006ST21:D5 (2009). Nanotechnology developments in India – a status report.

[136] United Nations (1995). Population and Development, Vol. 1: Programme of Action adopted at the International Conference on Population and Development, Cairo, 5–13 September 1994, paragraph 7.2.

[137] Guha SK (1996). Contraceptive for use by a male. US Patent 5,488,075.

[138] Guha SK (2007). Biophysical mechanism-mediated time-dependent effect on sperm of human and monkey vas implanted polyelectrolyte contraceptive. Asian J Androl.

[139] Stepanow S, Lin N, Vidal F, Landa A, Ruben M, Barth JV (2005). Programming supramolecular assembly and chirality in two dimensional dicarboxylate networks on a Cu(100) surface. Nano Lett.

[140] Guha SK (2005). RISUG™(reversible inhibition of sperm under guidance)–an antimicrobial as male vas deferens implant for HIV free semen. Med Hypotheses.

[141] Hauck TS, Giri S, Gao Y, Chan WCW (2010). Nanotechnology diagnostics for infectious diseases prevalent in developing countries. Adv Drug Deliv Rev.

[142] Usui Y, Aoki K, Murakami N, Nakamura I (2008). Carbon nanotubes with high bone-tissue compatibility and bone-formation acceleration effects. Small.

[143] Hilder TA, Hill JM (2009). Modeling the loading and unloading of drugs into nanotubes. Small.

[144] Lu F, Gu L, Meziani MJ, Wang X, Luo PG, Veca LM (2009). Advances in bioapplications of carbon nanotubes. Adv Mater.

[145] Liu Z, Cai W, He L, Nakayama N, Chen K, Sun X (2007). *In vivo* biodistribution and highly efficient tumour targeting of carbon nanotubes in mice. Nat Nanotechnol.

[146] Bhirde AA, Patel V, Gavard J, Zhang G, Sousa AA, Masedunskas A (2009). Targeted killing of cancer cells in Vivo and in Vitro with EGF-directed carbon nanotube-based drug delivery. ACS Nano.

[147] Hersam MC, Guisinger NP, Lyding JW (2000). Silicon-based molecular nanotechnology. Nanotechnology.

[148] Lapshin RV, Nalwa HS (2011). Feature-oriented scanning probe microscopy. Encyclopedia of nanoscience and nanotechnology.

[149] Cavalcanti A, Freitas RA, Kretly RC (2004). Nanorobotics control design: a practical approach tutorial.

[150] Latthe P, Latthe M, Say L, Gulmezoglu M, Khan KS (2006). WHO systematic review of prevalence of chronic pelvic pain: a neglected reproductive health morbidity. BMC Public Health.

